# Data independent acquisition reveals in-depth serum proteome changes in uremic pruritus

**DOI:** 10.3389/fphys.2024.1287072

**Published:** 2024-03-21

**Authors:** Zhao Wen-Jing, Tan Rui-Zhi, He Si-Yuan, Du Xiao-Mei, Hu Qiong-Dan, Zhang Xiao-Qian, Huang Wen-Hua, Su Hong-Wei, Liu Jian, Zhang Qiong, Wang Li

**Affiliations:** ^1^ Research Center of Intergated Traditional Chinese and Western Medicine, The Affiliated Traditional Chinese Medicine Hospital, Southwest Medical University, Luzhou, China; ^2^ Department of Nephrology, The Affiliated Hospital, Southwest Medical University, Luzhou, China; ^3^ School of Clinical Medicine, Southern Medical University, Guangzhou, China; ^4^ Department of Nephrology, The Affiliated Traditional Chinese Medicine Hospital, Southwest Medical University, Luzhou, China; ^5^ Department of Urology, The Affiliated Traditional Chinese Medicine Hospital, Southwest Medical University, Luzhou, China

**Keywords:** data independent acquisition, proteomics, uremic pruritus, CKD, DEPs

## Abstract

**Introduction:** Uremic pruritus (UP) is a prevalent symptom in patients suffering from uremia, yet its underlying etiology and mechanisms remain incompletely elucidated. Given the significant incidence of UP, identifying specific alterations in proteins present in the blood of UP patients could offer insights into the potential biological pathways associated with UP and facilitate the exploration of biomarkers.

**Methods:** In this study, we employed LC-MS/MS-based data-independent acquisition (DIA) mode to analyze serum samples obtained from 54 UP patients categorized as DKD-UP, HN-UP, and GN-UP (n = 18 for each subgroup), along with 18 uremic patients without pruritus (Negative) and 18 CKD patients without pruritus (CKD). Through DIA mode analysis, a total of 7075 peptides and 959 proteins were quantified. Within these, we identified four upregulated and 13 downregulated Differentially Expressed Proteins (DEPs) in DKD-UP versus Negative, five upregulated and 22 downregulated DEPs in HN-UP versus Negative, and three upregulated and 23 downregulated DEPs in GN-UP versus Negative. Furthermore, we conducted an intersection analysis of the DEPs across these three comparison groups to derive a set of common DEPs (COMP). Subsequently, a total of 67 common DEPs were identified in the three UP groups when compared to the CKD group, with 40 DEPs showing upregulation and 27 DEPs displaying downregulation.

**Results:** Following Gene Ontology (GO), Kyoto Encyclopedia of Genes and Genomes (KEGG), and Protein-Protein Interaction (PPI) analyses, we observed that the DEPs distinguishing UP from CKD were primarily associated with mitochondrial function (MT-CYB, PRDX2, TOMM22), inflammation (CD59, CSF1), renal injury (WFDC2), and neural function (CAP1, VGF).

**Discussion:** Our findings contribute to a potential molecular comprehension of UP pathogenesis, shedding light on the identification of these DEPs as plausible biomarkers for UP.

## Introduction

Uremic pruritus (UP) is a distressing symptom that commonly occurs in patients with end-stage renal disease (ESRD) (Mettang and Kremer, 2015). It is defined as a persistent and troublesome itch that can occur anywhere on the skin and is associated with scratching, dry skin, and changes in skin appearance (Combs et al., 2015; Rayner et al., 2017; Simonsen et al., 2017). The global prevalence of UP among hemodialysis patients has been reported to as high as 70% (Cheng and Wong, 2022). It is noteworthy that the prevalence of UP is particularly high in Asian regions, especially in China, Japan, and Pakistan, while it is the lowest in Germany (Zhang et al., 2023). UP affects the quality of life of patients with ESRD, leading to sleep disturbances, depression, and anxiety (Sukul et al., 2021; Titapiccolo et al., 2023). Given the high prevalence and negative impacts of UP, understanding its underlying mechanisms and developing effective treatments are urgent challenges.

Despite multiple hypotheses and recent progress in understanding how UP develops, its pathogenesis remains poorly understood. Uremic toxins, skin barrier impairments, neurogenic pathways, immune dysregulation, and other factors are thought to contribute to UP(Narita et al., 2008; Ko et al., 2023). Studies have indicated that low skin moisture in dialysis patients may be one of the contributing factors to induce pruritus (Morton et al., 1996; Ko et al., 2023; Krismi et al., 2023). Moreover, the significant increase in the number of dermal mast cells in UP patients is also considered as an important factor promoting skin itching (Robertson et al., 2016; Simonsen et al., 2017). Notably, immune dysregulation and inflammation are also crucial in inducing UP(Chen et al., 2010; Fallahzadeh et al., 2011; Ko et al., 2014). However, reports showed that most studies that examined the association between uremic toxins and UP have failed to replicate the findings of previous studies, suggesting the complexity of the underlying pathology of UP and the necessity for more research (Ozen et al., 2018; Verduzco and Shirazian, 2020).

Recent studies have shown that the blood proteomic has been widely used to explore the potential inflammatory and immune regulatory mechanisms underlying chronic skin itch diseases (Sutaria et al., 2022; Parthasarathy et al., 2023). It has also been beneficial in screening serum biomarkers for these diseases and the presence of disease endotypes (Parthasarathy et al., 2023). Currently, for UP, several potential protein biomarkers have been identified, such as C-reactive protein, substance P, interleukin two and interleukin 31 (Cho et al., 1997; Chen et al., 2010; Fallahzadeh et al., 2011; Oweis et al., 2021; Swierczynska et al., 2022). Among moderate/severe hemodialysis pruritic patients, those with higher high-sensitive C-reactive protein (hs-CRP) levels have higher mortality (Chen et al., 2010). Substance P, a neuropeptide mediator associated with pruritus in various dermatologic conditions, is also significantly higher in ESRD patients with pruritus (Cho et al., 1997). Another report demonstrated that the serum levels of IL-2 were significantly higher in hemodialysis patients with itch *versus* those without it, indicating the potential role of IL-2 in UP(Fallahzadeh et al., 2011). Interleukin 31, a proinflammatory cytokine produced by T helper two cells, can increase skin sensitivity to pruritogenic stimuli (Ko et al., 2014). Although these studies have shown significant differences in the expression of proteins in the blood of UP patients, they are only guided by existing differentially expressed proteins in skin itching caused by other triggers and cannot reveal new differentially expressed proteins and biomarkers, especially for the screening of itching related cellular markers and the revelation of related signaling pathways.

In this study, we plan to use Data Independent Acquisition (DIA) to reveal the serum proteomic changes in uremic pruritus patients, which may provide new avenues for studying UP, and may represent promising biomarkers for diagnosis as well as new targets for therapy.

## Materials and methods

### Clinical characteristics of patients and controls

Patients in this study included diabetic kidney disease-induced uremia with pruritus, hypertensive nephropathy-induced uremia with pruritus, glomerulonephritis-induced uremia with pruritus, uremia without pruritus, and chronic kidney disease (CKD) without pruritus. We collected serum (from January 2020 to June 2020) from a total of 90 patients for DIA proteomics analysis (including patients with diabetic kidney disease derived UP (DKD-UP), hypertensive nephropathy derived UP (HN-UP), glomerulonephritis derived UP (GN-UP), uremia without pruritus (Negative) and four to five stage CKD without pruritus (CKD), n = 18 for each group). We also recorded the information of these patients including gender, age, FIIQ score and some clinical indicators, blood pressure, creatinine, urea nitrogen, uric acid, cystatin C, plasma albumin, plasma globulin, triglyceride, cholesterol, low-density lipoprotein, high-density lipoprotein, aspartate aminotransferase, alanine aminotransferase and bilirubin. The degree of itching in patients is evaluated based on the four-item itch questionnaire (FIIQ), and only patients with severe itching are included (FIIQ = 11–15). All patients were tested for dialysis quality every 3 months, specifically for the Kt/V and URR parameters, which should be kept Kt/V > 1.2 and URR >70%. This study was approved by the Ethics Committee of Traditional Chinese Medicine Hospital Affiliated to Southwest Medical University (No: KY2020055).

### Total protein extraction from serum samples

Firstly, the free SDS lysate was added to 100 μL serum sample to make up the total volume of 1mL, and then DTT was added to the sample to a final concentration of 10 mM and incubated in the dark at 37°C for 30 min. Then iodoacetamide was added to the sample to a final concentration of 55 mM and incubated for another 30 min. The above protein mixture was enriched by solid phase extraction (SPE) C18 column to obtain dry protein. After dissolving the dried protein with 20 μL 50 mM ammonium bicarbonate solution, the peptide/protein was quantified according to the " Pierce Fluorescent Peptide Quantitative Determination Method " instructions and the polypropylene gel electrophoresis was performed to control the quality. The samples were separated by Shimadzu LC-20AB liquid phase system and 4.6 × 250 mm Gemini C18 column, and the components were obtained by freeze-drying. High pH RP separation was performed using Shimadzu LC-20AB HPLC system and Gemini high pH chromatographic column (5 m, 4.6 × 250 mm). The obtained components were combined, frozen and dried.

### DDA and DIA analysis by nano-LC-MS/MS

The DDA and DIA analysis were performed by BGI (Shenzhen, China). Briefly, the dried peptide samples were separated by Thermo UltiMate 3000 UHPLC. Firstly, the dried peptide sample was centrifuged at 20000 *g* for 10 min with mobile phase A (2% acetonitrile, 0.1% FA), and the supernatant was injected. The sample was first enriched and desalted by the trap column, and then connected in series with the self-installed C18 column (150 μm inner diameter, 1.8 μm column particle size, about 35 cm column length). The separation was carried out at a flow rate of 500 nL/min through the following effective gradient: 0–5 min, 5% mobile phase B (98% ACN, 0.1% FA); 5–90 min, the mobile phase B increased linearly from 5% to 25%; 90–105 min, mobile phase B increased from 25% to 35%; 105–110 min, mobile phase B increased from 35% to 80%; 110–115 min, 80% mobile phase B; 115–120 min, 5% mobile phase B. The peptides separated by liquid phase were ionized by nanoESI source and then entered into the tandem mass spectrometer Q-Exactive HF X (Thermo Fisher Scientific, San Jose, CA) for DDA (Data Dependent Acquisition) and DIA (Data Independent Acquisition) mode detection.

For DDA library building detection, the main parameter settings are as follows: the ion source voltage is set to 1.9 kV; the scan range of mass spectrometry was 350–1,500 m/z. The resolution was set to 120,000, and the maximum ion implantation time (MIT) was 50 m. The fragmentation mode of secondary mass spectrometry was HCD, and the fragmentation energy was set to NCE 28. The resolution is 30,000, the MIT is 100 m, and the dynamic exclusion time is set to 30 s. The initial m/z of the secondary mass spectrometry was fixed at 100; the parent ion screening conditions for the secondary fragmentation are: charge 2 + to 6 +, and the parent ion with peak intensity exceeding 2E4 is ranked in the top 20. AGC is set to: Level 1 3E6, Level 2 1E5.

For DIA mass spectrometry detection, the main parameters are set as follows: the ion source voltage is set to 1.9–2kV; the scanning range of primary mass spectrometry was 400–1250 m/z. The resolution is set to 120,000; the maximum ion implantation time (MIT) was 50 m. The 400–1250 m/z is divided into 45 windows for continuous window fragmentation and signal acquisition. The ion fragmentation mode is HCD, the MIT is in automatic mode, the fragment ions are detected in Orbitrap, the resolution is set to 30,000, and the fragmentation energy is distributed fragmentation: 22.5,25,27.5; AGC is set to 1E6.

### DDA data analysis

This project uses MaxQuant to identify DDA data and use it as a spectrum library for subsequent DIA analysis. The analysis uses the original data as the input file, and sets the corresponding parameters and database, and then performs identification and quantitative analysis. The identified peptides satisfying FDR ≤ 1% will be used to construct the final spectral library.

### DIA data analysis

The DIA data was analyzed using the iRT peptides for retention time calibration. Then, based on the target-decoy model applicable to SWATH-MS, false positive control was performed with FDR 1%, therefore obtaining significant quantitative results.

### MSstats differential analysis

The project used MSstats to statistically evaluate significant differences in proteins or peptides in different samples. The core algorithm is the linear mixed effect model. The process preprocesses the data according to a predefined comparison group, and then performs a significance test based on the model. After that, differential protein screening was performed using fold change >2 and *p*-value <0.05 as the criteria for significant differences. At the same time, an enrichment analysis of differential proteins was performed.

### ELISA

The level of cartilage oligomeric matrix protein (COMP) in serum of mice was detected by ELISA kit (Neobioscience, China). The absorbance of each sample was determined using a Synergy2 multi-function microplate reader (Bio-Tek, United States).

### Statistical analysis

The significance of differentially expressed proteins was evaluated based on a predefined control group and a linear mixed effect model. Two filtering criteria (fold change >2 and *p*-value <0.05) were used to obtain significant differentially expressed proteins. Euclidean distance and hierarchical clustering were used to cluster differential proteins. PPI analysis of DEPs was performed using the STRING (https://string-db.org) database). The interaction diagram was constructed using the first 100 interactions of confidence, and the diagram was drawn using Cytoscape 3.9.1 software. Functional enrichment analysis included gene ontology (GO) (http://www.geneontology.org) and Kyoto Encyclopedia of Genes and Genomes (KEGG) (http://www.genome.jp/kegg/pathway.html) data were visualized on the bioinformatic website (http://www.bioinformatics.com.cn). All statistical tests with *p* values less than 0.05 were considered statistically significant.

## Results

### Schematic illustration of the workflow used in this study

**FIGURE 1:**
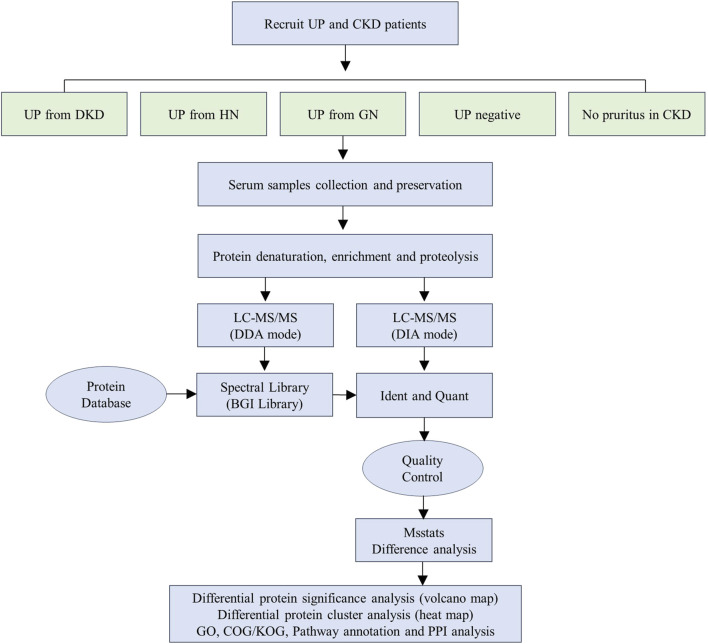
Schematic illustration of the workflow used in this study.

We performed DDA and DIA analyses using nanoLC-MS/MS to identify proteomic changes in serum of uremic pruritus patients. The workflow diagram illustrates the experimental process we established for this study (Figure 1). Initially, we recruited 90 patients and collected their serum samples. The study subjects comprised an equal number of patients with uremic pruritus derived from diabetic nephropathy (DKD-UP, n = 18), hypertensive nephropathy (HN-UP, n = 18), glomerulonephritis (GN-UP, n = 18), and uremic patients without pruritus (Neg, n = 18), as well as chronic kidney disease patients without pruritus (CKD, n = 18). Demographic information and clinical indicators are presented in Table 1. From each group, we randomly selected six patients and pooled their serum samples, resulting in a total of three samples for testing in each group. The samples were subjected to protein extraction, denaturation, enrichment, and enzymatic digestion, followed by nanoLC-mass spectrometry analysis using both DDA and DIA approaches. The identified proteins were matched with a protein database to determine specific protein sequences and amino acid compositions. After quality control, differentially expressed proteins were identified using MSstats differential analysis, and further analyzed through significance analysis, clustering analysis, and functional enrichment analysis.

**TABLE 1:** Clinical information of the patients involved in this study.

Index	Group				
	DKD-UP	HN-UP	GN-UP	Negative	CKD
Age (mean ± SD)	64.44 ± 10.01	58.67 ± 15.35	57.39 ± 12.45	62.44 ± 15.86	59.44 ± 11.79
FIIQ (mean ± SD)	12.89 ± 1.37	13.61 ± 1.34	13.33 ± 1.80	\	\
Blood Pressure (mean ± SD) (mmHg)	159.50 ± 23.20/86.14 ± 12.70	141.43 ± 30.46/84.36 ± 16.01	141.71 ± 26.86/83.43 ± 10.94	152.71 ± 15.54/80.43 ± 25.15	141.83 ± 22.77/83.44 ± 18.52
Cr (mean ± SD) (μmol/L)	921.27 ± 254.61	1035.53 ± 270.47	804.33 ± 367.35	825 ± 276.20	419.28 ± 355.51
BUN(mean ± SD) (mmol/L)	22.15 ± 4.96	20.02 ± 7.60	16.09 ± 7.38	18.68 ± 7.83	13.97 ± 8.95
Uric Acid (mean ± SD) (μmol/L)	458.6 ± 132.37	416.24 ± 122.57	402.73 ± 154.92	381.56 ± 141.29	345.94 ± 101.84
Cystatin c (mean ± SD) (mg/L)	7.89 ± 0.67	7.17 ± 1.66	6.60 ± 2.75	6.55 ± 2.26	4.41 ± 2.51
Plasma Albumin (mean ± SD) (g/L)	40.59 ± 6.60	38.01 ± 6.23	40.94 ± 5.18	39.28 ± 4.82	38.52 ± 6.30
Plasma Globulin (mean ± SD) (U/L)	26.59 ± 3.55	28.61 ± 6.32	26.75 ± 4.16	25.77 ± 4.06	29.21 ± 5.57
Triglyceride (mean ± SD) (mmol/L)	2.15 ± 1.38	2.37 ± 1.57	2.58 ± 1.12	2.34 ± 1.11	1.95 ± 1.07
Cholesterol (mean ± SD) (mmol/L)	3.22 ± 0.99	3.40 ± 0.61	3.69 ± 0.46	3.24 ± 0.73	4.64 ± 1.27
LDL (mean ± SD) (mmol/L)	1.52 ± 0.78	1.70 ± 0.55	1.84 ± 0.58	1.45 ± 0.56	2.53 ± 1.09
HDL (mean ± SD) (mmol/L)	0.76 ± 0.27	0.88 ± 0.21	1.03 ± 0.36	1 ± 0.28	1.27 ± 0.30
AST (mean ± SD) (U/L)	17.73 ± 8.77	16.31 ± 9.13	14.93 ± 7.53	15.63 ± 5.31	24.67 ± 12.42
ALT (mean ± SD) (U/L)	12.87 ± 8.19	10 ± 5.11	15.8 ± 11.87	13.69 ± 6.12	22.87 ± 10.14
Bilirubin (mean ± SD) (μmol/L)	7.39 ± 2.26	7.38 ± 1.13	7.34 ± 2.35	7.61 ± 2.27	7.23 ± 3.27

Note: SD: standard deviation; DKD-UP: diabetic kidney disease induced uremia with pruritus; HN-UP: hypertensive nephropathy induced uremia with pruritus; GN-UP: glomerulonephritis induced uremia with pruritus; Negative: Uremia without pruritus; CKD: chronic kidney disease without pruritus.

### Quality control and quantity of differential protein

**FIGURE 2:**
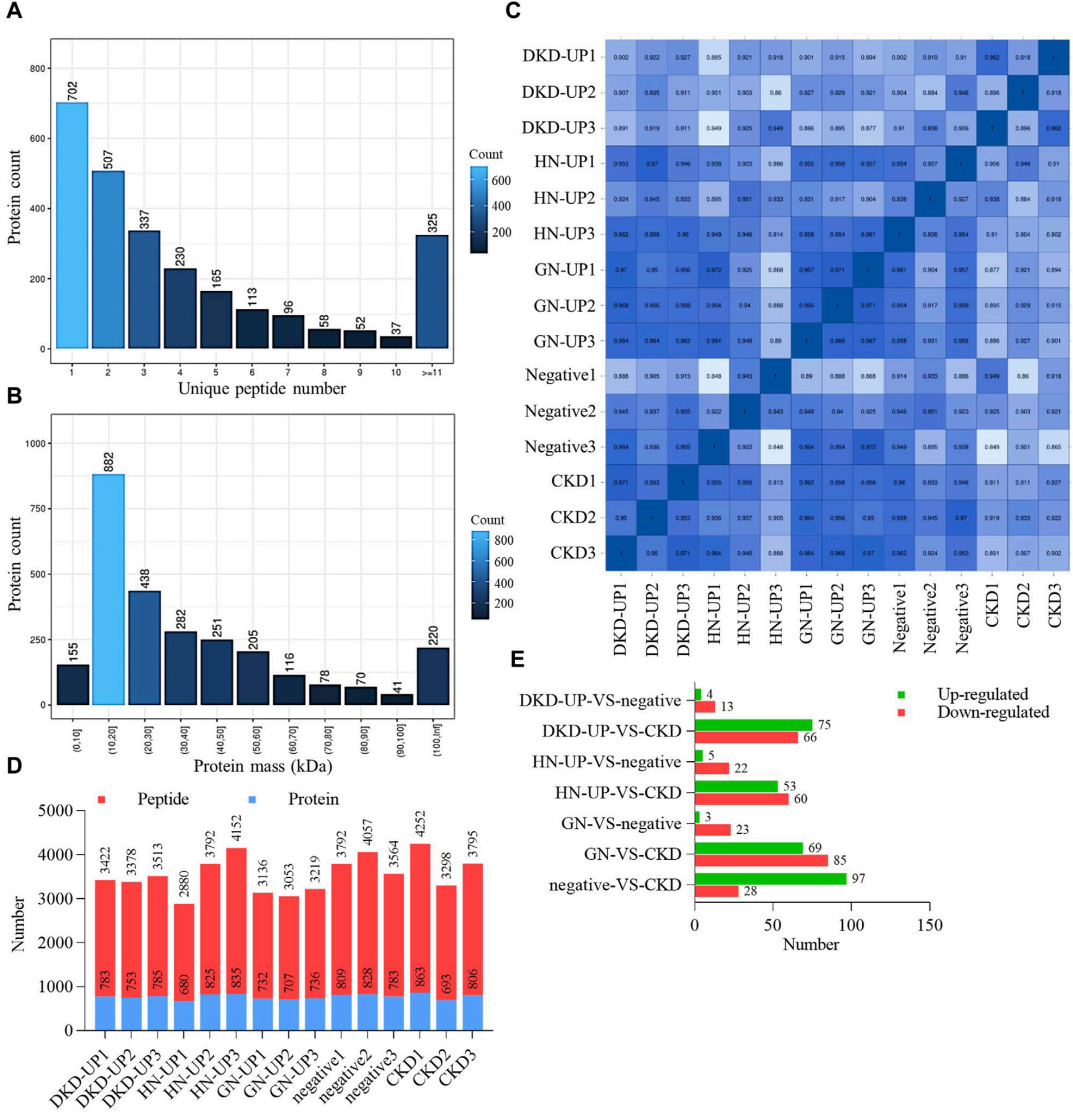
Quality control and quantity of differential protein. **(A)** The distribution of unique peptides identified by mass spectrometry. **(B)** Mass distribution of proteins identified by mass spectrometry. **(C)** Pearson’s correlation of protein quantitation. DKD-UP1, DKD-UP2, DKD-UP3: Serum samples from 18 DKD-UP patients were randomly pooled with every six samples combined into one group, resulting in the creation of three distinct groups designated as DKD-UP1, DKD-UP2, and DKD-UP3. The HN-UP group, GN-UP group, Negative group, and CKD group underwent a similar process. **(D)** The quantitative results of peptides and proteins identified in the 15 samples using mass spectrometry. **(E)** The number of upregulated and downregulated differential proteins in each comparison group.

We firstly collected dependent acquisition (DDA) mass spectrometry data from samples, and use MaxQuant to complete database search and identification, obtaining all detectable non-redundant high-quality MS/MS spectral information as the spectral library for subsequent data independent acquisition (DIA) quantification. Based on the results of DDA identification, we calculated the unique peptides number for each group and plotted a bar chart to visualize their distribution (Figure 2A). The number of unique peptides represents the confidence level of protein identification, with a higher number indicating higher confidence. We also analyzed the mass distribution range of the proteins, providing an overall view of the identified protein sizes. Approximately 92% of the proteins had a mass <100 kDa (Figure 2B). Finally, from the serum samples, we quantified a total of 7075 peptides and 959 proteins under DIA mode. To ensure data quality, we performed Pearson correlation analysis and displayed the expression correlations among samples in the form of a correlation matrix. The heatmap generated from the sample correlation analysis showed strong correlations among the 15 samples, indicating good reproducibility and statistical consistency (Figure 2C). Based on the quantitative results of peptides and proteins, the specific number of peptides and proteins in each sample is shown in Figure 2D. Additionally, we performed pairwise comparisons of serum proteins between each group. We applied two filtering criteria (fold change >2 and *p*-value <0.05) to identify proteins with significant differences (Figure 2E).

### Differentially expressed proteins of UP groups to UP negative group

**FIGURE 3:**
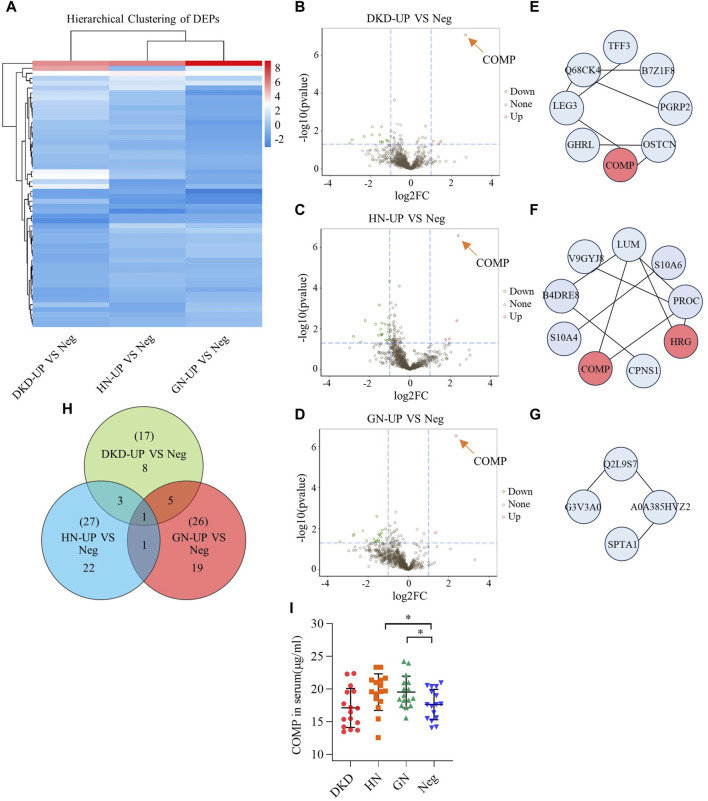
Differentially Expressed Proteins of UP groups to UP negative group. **(A)** DEPs clustering heat map of DKD-UP group, HN-UP group and GN-UP group compared with Neg group. **(B)** DEPs volcano plot of DKD-UP group compared with Neg group. **(C)** DEPs volcano plot of HN-UP group compared with Neg group. **(D)** DEPs volcano plot of GN-UP group compared with Neg group. **(E)** Protein-protein network analysis of DEPs between DKD-UP group and Neg group. **(F)** Protein-protein network analysis of DEPs between HN-UP group and Neg group. **(G)** Protein-protein network analysis of DEPs between GN-UP group and Neg group. **(H)** The Venn diagram of the number of DEPs identified in the three comparison groups (DKD-UP VS Neg, HN-UP VS Neg, GN-UP VS Neg). **(I)** The COMP content in serum of four study groups (DKD-UP, HN-UP, GN-UP and Neg) were detected by enzyme-linked immunosorbent assay (ELISA). *p < 0.05.

In order to further analyze the proteomic differences in the serum of patients with uremic pruritus and uremic non pruritus, hierarchical clustering was used to analyze the Differentially Expressed Proteins (DEPs), and the results were presented in a heatmap (Figure 3A). A total of three groups of DEPs were obtained by comparing the serum proteins of three groups of uremic pruritus patients with those of uremic patients without pruritus. In comparison to the Neg group, the DKD-UP group showed upregulation of 4 DEPs and downregulation of 13 DEPs; the HN-UP group showed upregulation of 5 DEPs and downregulation of 22 DEPs; and the GN-UP group showed upregulation of 3 DEPs and downregulation of 23 DEPs, indicating distinct proteomic patterns between patients with uremic pruritus and those without pruritus. The significantly altered proteins in the four groups of plasma were visualized using a volcano plot (Figures 3B–D) and presented in Table 2. We also analyzed specific protein-protein interactions (PPIs) among the DEPs in each group, and some of them were found to be involved in the regulation of the PPI network (Figures 3E–G). In the comparison between the DKD-UP and Neg groups, it was found that Galectin-3 (LEG3) and Leucine-rich alpha-2-glycoprotein (Q68CK4) had the highest degree of interaction in the PPI network, followed by Cartilage Oligomeric Matrix Protein (COMP) and Osteocalcin (OSTCN). In the comparison between the HN-UP and Neg groups, Lumican (LUM) and Protein C (PROC) showed the highest degree of interaction in the PPI network, followed by COMP, cDNA FLJ58 (B4DRE8), and Histidine-rich Glycoprotein (HRG). In the comparison between the GN-UP and Neg groups, Mutant Hemoglobin Subunit Alpha-2 (A0A385HVZ2) and Alpha-1-Antitrypsin M Brescia variant (Q2L9S7) showed the highest degree of interaction in the PPI network, followed by Spectrin Alpha Chain, Non-erythrocytic 1 (SPTN1), Serpin Family A Member 3 (G3V3A0), cDNA FLJ58164 (B4DRE8), and HRG. Additionally, we analyzed and visualized the DEPs in each group of uremic pruritus patients and uremic patients without pruritus using a Venn diagram, and found a common DEP, COMP (Figure 3H). Finally, we determined the levels of COMP in the serum of patients in each group using an enzyme-linked immunosorbent assay (ELISA) (Figure 3I). The results showed that COMP significantly increased in the serum of HN-UP and GN-UP patients.

**TABLE 2:** The up- and downregulated DEPs in DKD-UP group, HN-UP group and GN-UP group compared with Neg group.

	UP				Down							
DKD-UP VS Neg	COMP	LTBP4	Q0ZCJ1	Q6ZSD0	HV338	OSTCN	IGD	NDKA	LEG3	TFF3	PGRP2	
					AHSP	GHRL	A0A5C2GH32	B7Z1F8	Q0KKI6	Q68CK4		
HN-UP VS Neg	HRG	COMP	A0A5C2G7J3		PROC	CPNS1	S10A6	MGP	SPRC	ELN	SIAT1	S10A4
	A0A5C2GHB5	A0A5C2GKY4			LUM	MYL6	TAGL	TFF3	GUC2B	FAM3C	AHSP	A0A0A7C4E4
					A0A1L2BU51	B4DRE8	Q0KKI6	Q5XTR9	Q6GMV7	V9GYJ8		
GN-UP VS Neg	COMP	PCM1	Q6ZSD0		KIF5C	SPTA1	C1QA	IGD	ELN	NDKA	WAC2A	PGRP2
					A0A2U3U020	A0A2Y9CYD6	A0A385HVZ2	A0A5C2FVM9	A0A5C2FZF6	A0A5C2GMW8	A0A5C2GSA3	A2NV54
					G3V3A0	Q0ZCJ1	Q2L9S7	Q5NV91	Q7Z351	S6B2C3	S6C4S2	

Note: DKD-UP: diabetic kidney disease induced uremia with pruritus; HN-UP: hypertensive nephropathy induced uremia with pruritus; GN-UP:glomerulonephritis induced uremia with pruritus; Negative: Uremia without pruritus; CKD: chronic kidney disease without pruritus.

### The GO and pathway analysis of differential proteins in UP groups to UP negative group

**FIGURE 4:**
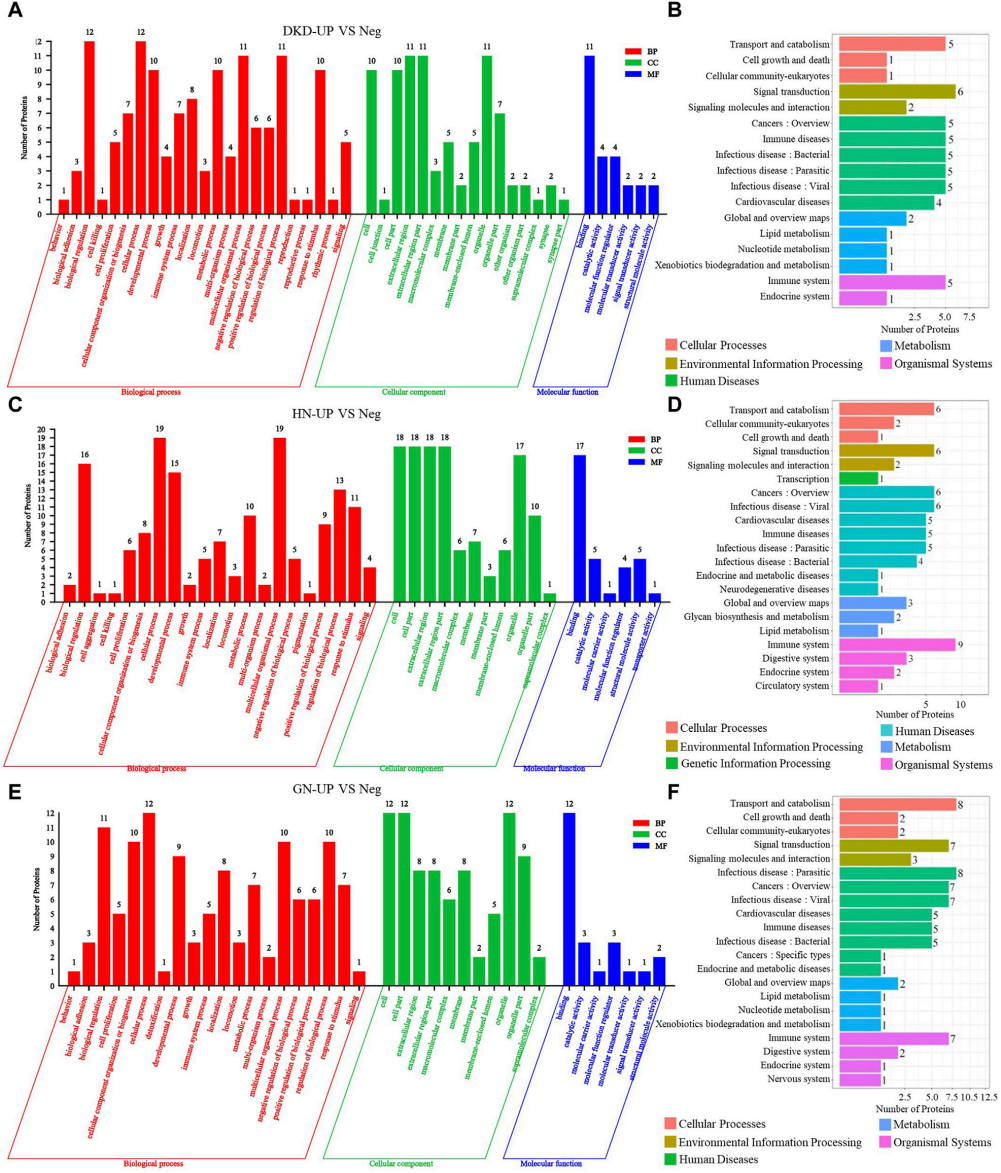
The GO and pathway analysis of differential proteins in UP groups to UP negative group. **(A,C and E)** The enrichment analysis of differentially expressed proteins between UP groups and Negative group based on GO, including biological process (BP), cellular component (CC) and molecular function (MF). **(B,D and F)** DEPs enrichment analysis based on KEGG pathway was performed in UP groups and Negative group.

We then performed gene ontology (GO) analysis on the DEPs from each comparison group. In the comparison between DKD-UP and Neg groups, GO functional enrichment analysis revealed 45 GO terms, including 23 related to biological processes, 16 related to cellular components, and six related to molecular functions. The GO enrichment bar chart showed that the main biological processes involved biological regulation, cellular processes, multicellular organism processes, and regulation of biological processes. The cellular components included extracellular region, extracellular region part, and organelles. The molecular functions involved binding, catalytic activity, and molecular function regulators (Figure 4A). KEGG pathway enrichment analysis identified 17 enriched pathways, with signal transduction being the most significant, followed by transport and catabolism, cancers, immune disease and infectious disease (Figure 4B). In the comparison between HN-UP and Neg groups, GO functional enrichment analysis revealed 38 GO terms, including 21 related to biological processes, 11 related to cellular components, and six related to molecular functions. The GO enrichment bar chart showed that the main biological processes involved multicellular organism processes, cellular processes, biological regulation, and developmental processes. The cellular components included cells, cell components, extracellular region, and extracellular region part. The molecular functions involved binding, catalytic activity, and structural molecular activity (Figure 4C). KEGG pathway enrichment analysis identified 21 enriched pathways, with the immune system pathway being the most significant, followed by transport and catabolism, signal transduction, cancers, and infectious disease pathways (Figure 4D). In the comparison between GN-UP and Neg groups, GO functional enrichment analysis revealed 38 GO terms, including 20 related to biological processes, 11 related to cellular components, and seven related to molecular functions. The GO enrichment bar chart showed that the main biological processes involved cellular processes, biological regulation, cellular component organization or biogenesis, multicellular organism processes, and regulation of biological processes. The cellular components included cells, cell components, and organelles. The molecular functions involved binding, catalytic activity, and molecular function regulators (Figure 4E). KEGG pathway enrichment analysis identified 21 enriched pathways, with transport and catabolism and infectious disease pathways being the most significant, followed by signal transduction, cancers, and immune system pathways (Figure 4F). These results showed that the GO and signal pathways enrichment involved differential proteins between patients with and without pruritus after uremia induced by different reasons.

### Differentially expressed proteins of UP groups to CKD group

**FIGURE 5:**
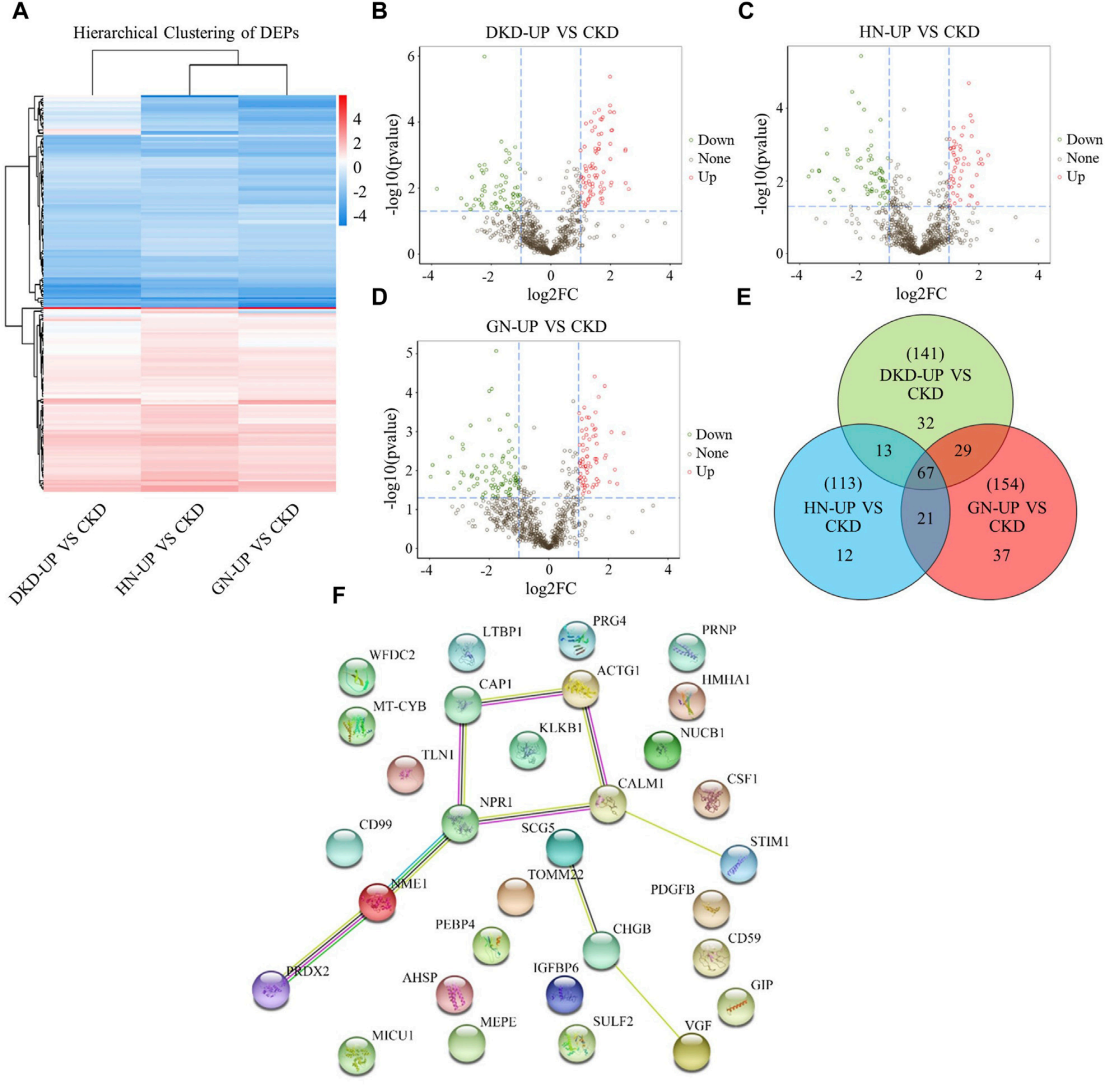
Differentially Expressed Proteins of UP groups to CKD group. **(A)** DEPs clustering heat map of DKD-UP group, HN-UP group and GN-UP group compared with CKD group. **(B)** DEPs volcano plot of DKD-UP group compared with CKD group. **(C)** DEPs volcano plot of HN-UP group compared with CKD group. **(D)** DEPs volcano plot of GN-UP group compared with CKD group. **(E)** The Venn diagram of the number of DEPs identified in the three comparison groups (DKD-UP VS CKD, HN-UP VS CKD and GN-UP VS CKD). **(F)** Protein-protein network interaction diagram of intersection proteins between UP groups and CKD group.

We further compared the differences in serum proteins between patients with uremic pruritus and those with CKD in stages 4–5 without pruritus. The DEPs were analyzed using hierarchical clustering, and the results are presented as a Heatmap (Figure 5A). By comparing the serum proteins of three uremic pruritus groups with those of CKD non-pruritus patients, we obtained three sets of proteins with upregulated or downregulated expression. Compared to the CKD group, the DKD-UP group had 75 upregulated DEPs and 66 downregulated DEPs; the HN-UP group had 53 upregulated DEPs and 60 downregulated DEPs; the GN-UP group had 69 upregulated DEPs and 85 downregulated DEPs, indicating distinct proteomic patterns between uremic pruritus patients and CKD non-pruritus patients. The volcano plots were used to visualize the significantly altered proteins in the four groups of serum samples (Figures 5B–D). We then analyzed and visualized the DEPs in each group of uremic pruritus patients and CKD non-pruritus patients using a table and a Venn diagram, and found that there are 67 common differentially DEPs (Table 3; Figure 5E). Furthermore, we analyzed specific protein-protein interactions (PPIs) among the intersecting DEPs in these three comparison groups, and some of them were involved in the regulation of PPI networks (Figure 5F). The above results indicated we detected more differential proteins in the serum of uremia patients with pruritus and CKD patients without pruritus, which may provide key evidence for exploring the potential pathogenesis of uremia pruritus.

**TABLE 3:** The up- and downregulated DEPs in DKD-UP group, HN-UP group and GN-UP group compared with CKD group.

	UP								Down					
DKD、HN、GN-UP VS CKD	VGF	TGON2	CILP1	PDGFB	CALC	PRPC	CYTB	SCG1	SC22B	KLKB1	CPNS1	S10A6	CAN1	CALM1
	CSF1	GIP	RNAS2	OSTP	CMGA	CD59	CD99	IBP6	SYUA	F10A1	ACTG	TBA1B	CAP1	STIM1
	GUC2A	NUCB1	AHNK	NAR3	WFDC2	LTBP1	SULF2	PRG4	A0A1S5UZ39	A0A385HVZ2	A0A5C2G196	Q13707	Q4TZM4	Q5XTR9
	ISK5	MEPE	TOMM22	EMIL1	A0A384NL10	B4DEA7	B4DRW4	C9IYI1	NDKA	COF1	S10A4	PRDX2	HMHA1	PDLI7
	7B2	HEP2	CAD13	PGBM	PEBP4	EMIL2	F4YU93	Q6LBZ0	AHSP	TLN1	Q9BWU5			

Note: DKD-UP: diabetic kidney disease induced uremia with pruritus; HN-UP: hypertensive nephropathy induced uremia with pruritus; GN-UP:glomerulonephritis induced uremia with pruritus; Negative: Uremia without pruritus; CKD: chronic kidney disease without pruritus.

### The GO and pathway analysis of differential proteins in respective UP group to CKD group

**FIGURE 6:**
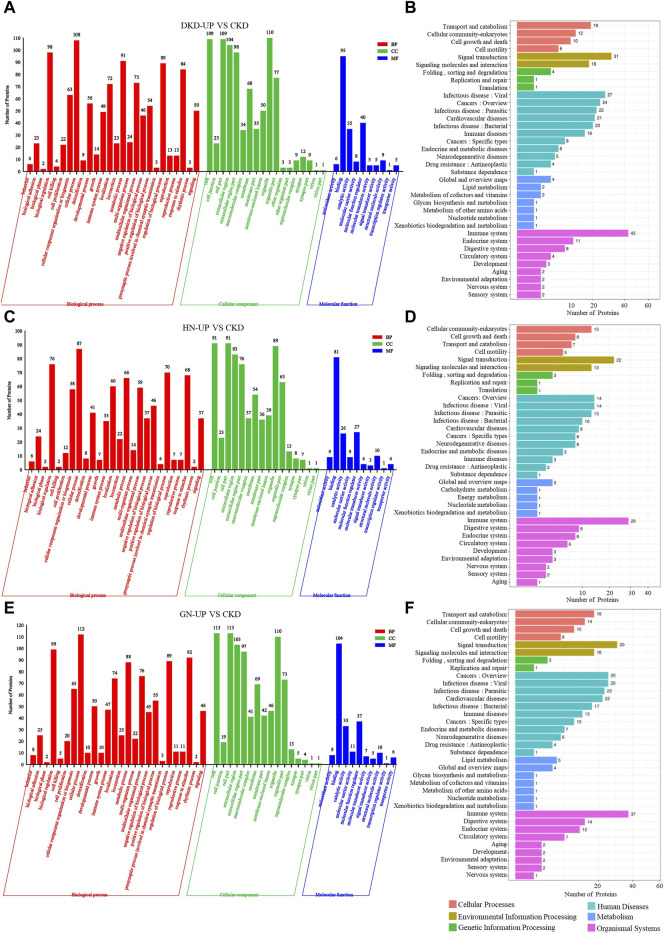
The GO and pathway analysis of differential proteins in respective UP group to CKD group. **(A,C and E)** The enrichment analysis of differentially expressed proteins between UP groups and CKD group based on GO, including biological process (BP), cellular component (CC) and molecular function (MF). **(B,D and F)** DEPs enrichment analysis based on KEGG pathway was performed in UP groups and CKD group.

We further conducted GO and KEGG pathway enrichment analysis on the DEPs from each comparison group. In the comparison between DKD-UP and CKD groups, GO functional enrichment analysis identified 54 GO terms, including 26 related to biological processes, 18 related to cellular components, and 10 related to molecular functions. The GO enrichment bar chart revealed that the main biological processes involved were cellular processes, biological regulation, metabolic processes, and regulation of biological processes. The cellular components included organelles, cells, cellular components, and extracellular regions, while the molecular functions included binding, molecular function modulators, and catalytic activity (Figure 6A). KEGG pathway enrichment analysis identified 36 enriched pathways, with the immune system being the most significant pathway, followed by signal transduction, infectious disease, cancers and cardiovascular diseases (Figure 6B). After comparing the HN-UP group and CKD group, GO functional enrichment analysis resulted in 52 GO terms, including 26 terms related to biological processes, 16 terms related to cellular components, and 10 terms related to molecular functions. A bar graph was generated to represent the enriched GO terms. The main biological processes involved were cell processes, biological regulation, regulation of biological processes, and response to stimuli. The cellular components involved were cells, cell parts, organelles, and extracellular regions. The molecular functions involved were binding, catalytic activity, and molecular function regulation (Figure 6C). In addition, the KEGG pathway enrichment analysis identified 34 pathways. The most significant pathway was also the immune system, followed by signal transduction, cancers and infectious diseases (Figure 6D). For comparing the GN-UP group and CKD group, GO functional enrichment analysis resulted in 52 GO terms, including 26 terms related to biological processes, 16 terms related to cellular components, and 10 terms related to molecular functions. The main biological processes involved were cell processes, biological regulation, regulation of biological processes, and metabolic processes. The cellular components involved were cells, cell parts, organelles, and extracellular regions. The molecular functions involved were binding, molecular function regulators, catalytic activity, and molecular carrier activity (Figure 6E). In addition, the KEGG pathway enrichment analysis identified 34 pathways. The most significant pathway was the immune system, followed by signal transduction, cancers, infectious diseases and cardiovascular diseases (Figure 6F).

### GO and KEGG analysis of common differential proteins in three UP groups compared to CKD group

**FIGURE 7:**
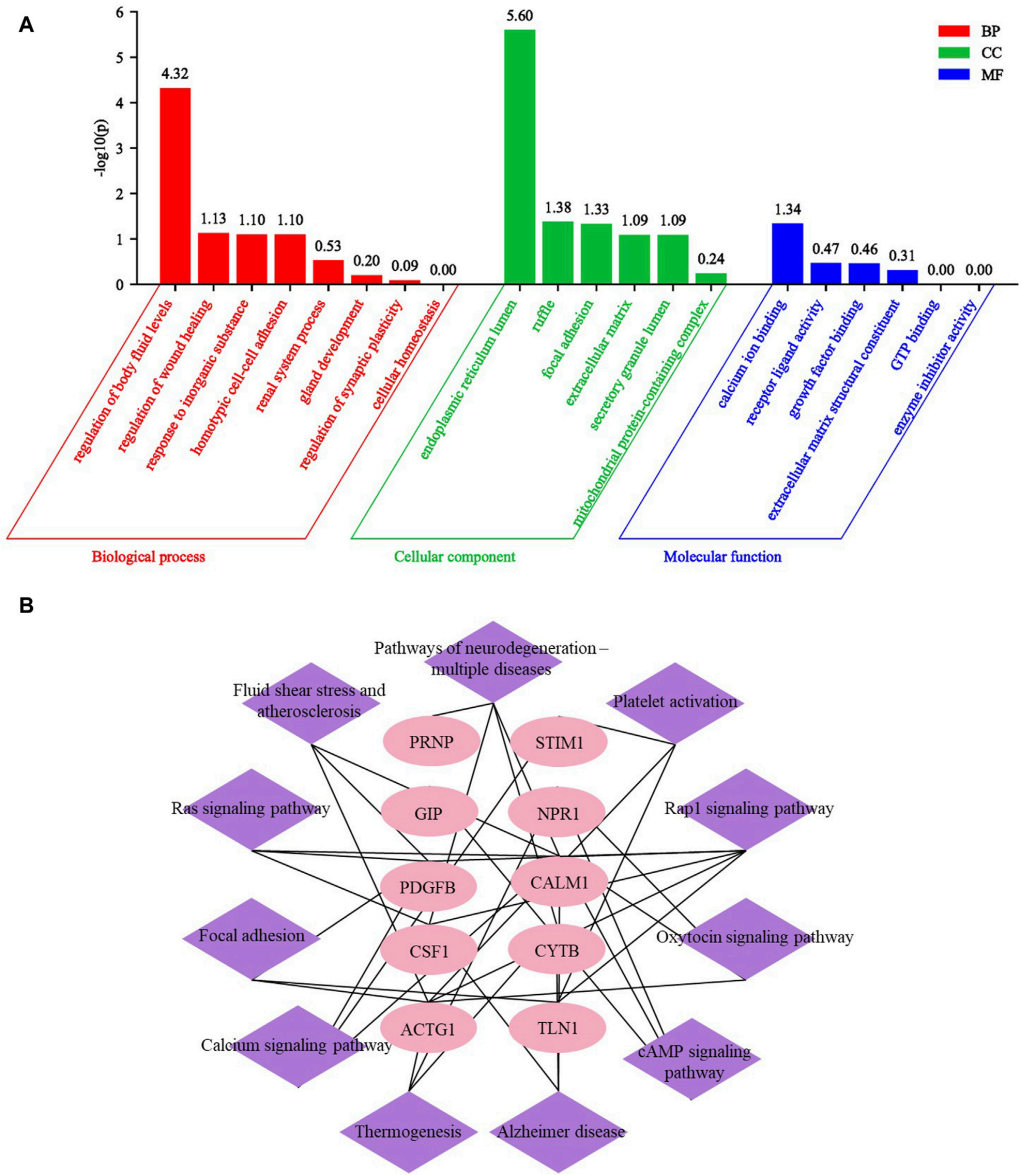
GO and KEGG analysis of common differential proteins in three UP groups compared to CKD group. **(A)** Enrichment GO analysis of common differential proteins of UP groups and CKD group. **(B)** The KEGG pathway target map of DEPs after comparison between UP groups and CKD group.

After conducting GO and KEGG analysis on differential proteins between respective UP groups and CKD non pruritus group, we subsequently conducted GO and KEGG analysis on the 67 common DEPs after comparing the three UP groups and CKD non pruritus group. The GO analysis identified 20 GO terms and 11 KEGG pathways. Among the GO terms, eight were related to biological processes, six were related to cellular components, and six were related to molecular functions. A bar graph was generated to represent the enriched GO terms. The main biological processes involved were regulation of fluid levels, regulation of wound healing, response to inorganic substances, and homotypic cell-cell adhesion. The cellular components involved were endoplasmic reticulum lumen, focal adhesion, and extracellular matrix. The molecular functions involved were calcium ion binding, receptor ligand activity, and growth factor binding (Figure 7A). The Rap1 signaling pathway was the most significant KEGG pathway, followed by fluid shear stress and atherosclerosis, and the ras signaling pathway. Based on the enriched pathways and their corresponding target proteins, a pathway-target network was constructed between 10 important proteins and 11 pathways (Figure 7B). These results can reveal the serum differential proteins between UP patients and CKD non pruritus patients and the interaction of the related pathways.

## Discussion

The exact mechanism of pruritus in uremic patients is not yet fully understood. Although several theories, such as the accumulation of uremic toxins and inflammatory processes, have been proposed, further research is still needed to elucidate the underlying mechanisms. Currently, there are no specific biomarkers available to accurately diagnose or monitor uremic pruritus. Given that certain treatments may manifest their effects through the inhibition of biomarker activity, which patients may not perceptibly discern in clinical settings, it is of paramount importance to comprehensively assess both biomarkers and patients’ subjective reports of symptom changes in appraising the efficacy of therapeutic interventions. On this basis, the screening of serum proteins in uremic patients with pruritus may be a promising approach to identify biomarkers and explore the underlying biological mechanisms of uremic pruritus. In this study, we collected serum samples from 54 of uremic patients with pruritus (including 18 patients with diabetic nephropathy progressing to uremia with pruritus, 18 patients with hypertensive nephropathy progressing to uremia with pruritus, and 18 patients with glomerulonephritis progressing to uremia with pruritus), as well as 18 of uremic patients without pruritus and 18 of stage 4–5 chronic kidney disease (CKD) patients without pruritus. Through DIA quantitative analysis of the differential proteins in the serum of pruritic patients compared to uremic patients and CKD patients, the aim was to identify potential biomarkers and explore the potential cellular and molecular mechanisms related to this disease.

For a long time, research on the pathogenesis of uremic pruritus has mainly focused on metabolism and toxic substances. However, these factors may only serve as triggering elements for uremic pruritus, and the underlying biological mechanisms remain unclear. In particular, the action cells, cytokines, and molecular signaling pathways involved in the development of uremic pruritus have not been studied. Therefore, we collected serum samples from patients who developed uremia and subsequently experienced pruritus induced by different factors, and performed protein DIA quantitative analysis comparing their serum to that of uremic patients without pruritus. Additionally, we collected serum samples from stage 4–5 CKD patients without pruritus and compared their serum protein profiles with those of uremic pruritus patients. The aim was to explore the proteomic changes associated with itch induction in patients who develop pruritus after entering uremia, compared to CKD patients who do not develop uremic pruritus. First, we performed the comparison of serum DEPs between UP patients and uremia without pruritus patients, 17 DEPs were detected between the DKD-UP group and Neg group, including four upregulated DEPs and 13 downregulated DEPs. For the HN-UP group compared to the Neg group, 27 DEPs were detected, with five upregulated and 22 downregulated. As for the GN-UP group compared to the Neg group, 26 DEPs were detected, with three upregulated and 23 downregulated. Notably, only COMP showed significant upregulation in all uremic pruritus groups, and this result was subsequently confirmed by ELISA. It is worth mentioning that the limited number of differential proteins may be due to the homogeneity of serum samples from uremic patients or insufficient sample size. COMP is a large pentameric glycoprotein that interacts with multiple extracellular matrix proteins in cartilage and other tissues (Posey et al., 2018). Previous studies have found that COMP is associated with collagen secretion and fibrogenesis (Agarwal et al., 2012), chondrocyte proliferation, and maintenance of tendon mechanical strength (Sodersten et al., 2005). In clinical practice, COMP is regarded as a biomarker for idiopathic pulmonary fibrosis and cartilage degeneration, as well as a prognostic marker for joint damage in rheumatic diseases (Posey et al., 2018). Studies have shown that COMP is induced by TGF-β stimulation, and time-dependent increases in COMP mRNA and protein levels were observed through PCR, Western blot, and ELISA after stimulation with TGF-β1 (5 ng/mL) (Vuga et al., 2013). Immunofluorescence staining of phosphorylated Smad3 (p-Smad3) and COMP revealed co-localization of COMP protein with p-Smad3 (Vuga et al., 2013). Interestingly, reports suggested that the Smad3 pathway can regulate allergen-induced skin inflammation and systemic IgE antibody production in a mouse model of atopic dermatitis (Anthoni et al., 2007), indicating that the Smad3 signaling pathway may be a key signaling target in allergic skin diseases (Shafi et al., 2023). In light of our DIA results, the high expression of COMP in the serum of uremic pruritus patients may promote itch occurrence through its interaction with Smad3. Furthermore, COMP also has the potential to serve as a biomarker for uremic pruritus. However, these hypotheses still need to be further validated by increasing the sample size and conducting experimental verification.

Subsequently, we compared the DEPs in the serum of uremic pruritus patients and CKD patients without pruritus, which plays a crucial role in understanding the potential mechanisms of uremia-induced itch. Data analysis revealed 141 DEPs between the DKD-UP and CKD groups, with 75 upregulated and 66 downregulated. In the comparison between the HN-UP and CKD groups, 113 DEPs were found, with 53 upregulated and 60 downregulated. As for the comparison between the GN-UP and CKD groups, 154 DEPs were identified, with 69 upregulated and 85 downregulated. GO analysis showed that these DEPs were mainly associated with cellular processes, biological regulation, extracellular region, and binding. KEGG analysis revealed that these DEPs were mainly related with transport and catabolism, signal transduction, infectious diseases, and the immune system. According to protein-protein interaction (PPI) and functional analysis, we found that a large number of DEPs involved in mitochondrial function, inflammation, kidney injury and fibrosis, as well as neural function. For example, the antioxidant protein Prdx2 showed significant downregulation in all pruritus groups (Li et al., 2020; Wang et al., 2020). Tomm22 protein, a core component of the mitochondria outer membrane protein translocation pore, is the human TOM complex and the proapoptotic protein Bax receptors, that has been found upregulated in all pruritus groups (Bellot et al., 2007; Su et al., 2022). Mitochondrially encoded cytochrome B (cytochrome b, MT-CYB or CYTB), a member of the oxidative phosphorylation system, can affect the production of free radicals, which is also upregulated in all pruritus groups (Borek et al., 2016; Pirola et al., 2021). The identification of these DEPs related to mitochondrial function suggests that uremic pruritus may significantly relate to oxidative stress and mitochondrial dysfunction, providing insights into the cellular and molecular mechanisms of UP. Inflammation is also considered an important triggering factor in uremic pruritus. Our results revealed a significant number of DEPs that are involved in inflammation. Cd59, a small GPI- anchored glycoprotein, it is developmental inflammation is a key factor in the development of myelinating glial cells (Wiltbank et al., 2022). In addition, the internalization of CD59, which acts as a complement inhibitor, can increase endothelial inflammation (Emin et al., 2016). Another DEPs associated with inflammation is CSF1, a protein involved in the homeostasis and inflammation of macrophages (Lin et al., 2019). These proteins suggest potential links between uremic pruritus and inflammation, providing data for research on the inflammatory processes involved in uremic pruritus. Interestingly, we found a significant increase in the level of WFDC2 protein (also known as HE4) in all uremic pruritus groups compared to the CKD group. This protein has been reported as a biomarker for the severity of kidney disease and fibrosis (Wan et al., 2016; Luo et al., 2018). Furthermore, abnormal neuronal signaling is also considered an important factor in uremic pruritus. Our results indicate that CAP1 is downregulated in the uremic pruritus groups, which has been reported to play crucial roles in growth cone function, neuron differentiation, and neuron connectivity in the brain (Schneider et al., 2021). Another protein that showed increased expression is VGF, which has been shown to be a biomarker and target for neurodegenerative and psychiatric disorders (Quinn et al., 2021). On the other hand, uremic pruritus is frequently linked to a heightened cardiovascular mortality rate among patients with CKD (Tonelli et al., 2016). Subsequently, we examined the correlation of DEPs with cardiovascular incidents. For instance, the STIM1 protein was observed to be present in reduced quantities in individuals with UP, and prior research indicates that silencing of Stim1 augments the activity of the anti-hypertrophic and pro-apoptotic molecule GSK-3β, which may accelerate the progression towards heart failure (Benard et al., 2016). This suggests that a reduction of STIM1 in UP patients may have a correlation with the development of cardiac failure. Additionally, the protein CALM1 was also found in decreased concentrations within the serum of UP patients. Mutations in this protein have been associated with an increased risk of sudden cardiac death in Chinese patients of Han ethnicity with chronic cardiac failure (Liu et al., 2015). This further suggests that diminished expression of CALM1 may elevate the risk of sudden cardiac death in the affected individuals. Collectively, these findings propose that the differential serum proteins in UP patients could be implicated in cardiovascular events.

While more and more research teams are starting to focus on the mechanisms and treatment of uremic pruritus, there has always been a limitation in the advancement of uremic pruritus research. That is the lack of a widely recognized and effective experimental animal model for uremic or CKD-related pruritus. This limitation hinders further exploration of the specific functions of these above proteins in uremic pruritus. However, our results may potentially provide candidate molecules for the establishment of an effective animal model for uremic pruritus.

## Conclusion

Taken together, based on DIA protein quantification analysis, this study identified differentially expressed proteins in the serum of uremic pruritus patients compared to non-pruritus uremic patients. It also discovered DEPs in the serum of uremic pruritus patients compared to CKD patients. Analysis of these DEPs provided insights into their potential involvement in various biological functions and underlying molecular signaling pathways, thus providing key data for the selection of biomarkers for uremic pruritus and understanding the underlying cellular and molecular biology mechanisms.

## Data Availability

The original contributions presented in the study are publicly available. This data can be found here: The mass spectrometry proteomics data have been deposited to the ProteomeXchange Consortium via the PRIDE partner repository, http://www.ebi.ac.uk/pride/archive/projects/PXD045336.
